# Stress Increases Peripheral Axon Growth and Regeneration through Glucocorticoid Receptor-Dependent Transcriptional Programs

**DOI:** 10.1523/ENEURO.0246-17.2017

**Published:** 2017-08-21

**Authors:** Jessica K. Lerch, Jessica K. Alexander, Kathryn M. Madalena, Dario Motti, Tam Quach, Akhil Dhamija, Alicia Zha, John C. Gensel, Jeanette Webster Marketon, Vance P. Lemmon, John L. Bixby, Phillip G. Popovich

**Affiliations:** 1 Center for Brain and Spinal Cord Repair, Department of Neuroscience, Wexner Medical Center at the Ohio State University, Columbus, OH 43210; 2 The Miami Project to Cure Paralysis, Department of Neurological Surgery, The University of Miami, Miami, FL 33136; 3 Department of Pharmacology, The University of Miami, Miami, FL 33136; 4 Center for Gene Therapy, The Research Institute at Nationwide Children's Hospital, Columbus, OH 43205; 5 Spinal Cord and Brain Injury Research Center, Department of Physiology, The University of Kentucky, Lexington, KY 40536; 6 SRA International Inc, Beavercreek, OH 45431

**Keywords:** *Key Words*: dorsal root ganglia, glucocorticoid receptor, plasticity, stress

## Abstract

Stress and glucocorticoid (GC) release are common behavioral and hormonal responses to injury or disease. In the brain, stress/GCs can alter neuron structure and function leading to cognitive impairment. Stress and GCs also exacerbate pain, but whether a corresponding change occurs in structural plasticity of sensory neurons is unknown. Here, we show that in female mice (*Mus musculus*) basal GC receptor (*Nr3c1*, also known as GR) expression in dorsal root ganglion (DRG) sensory neurons is 15-fold higher than in neurons in canonical stress-responsive brain regions (*M. musculus*). In response to stress or GCs, adult DRG neurite growth increases through mechanisms involving GR-dependent gene transcription. *In vivo*, prior exposure to an acute systemic stress increases peripheral nerve regeneration. These data have broad clinical implications and highlight the importance of stress and GCs as novel behavioral and circulating modifiers of neuronal plasticity.

## Significance Statement

Nerve injury-induced pain affects millions and is a comorbidity factor for individuals living with traumatic spinal cord or peripheral nerve injuries. Pain is associated with aberrant plasticity and sprouting in the injured peripheral and central nervous systems. However, the mechanisms underlying these structural changes are not understood. Here, new data implicate stress hormones (steroids) and GR activation as a novel mechanism underlying enhanced sensory neuron plasticity in injured peripheral nerves. These data also have important implications for the development and care of nerve-injury induced pain, including the use of steroids as a treatment for inflammatory pain.

## Introduction

Stress, a ubiquitous and recurrent part of daily life, activates complex regulatory mechanisms in the body that cause glucocorticoids (GCs) to increase in the circulation. GCs are steroid hormones that dramatically affect cell function. They do so mainly by binding to GC receptors (GRs), which act in the nucleus to regulate gene transcription. In the brain, neurons in the cerebral cortex and subcortical structures (e.g., hippocampus, amygdala) express high levels of GRs (Sapolsky et al., 1984) and GCs/stress can modulate their structural integrity. For example, GCs cause dendritic hypertrophy in amygdala neurons (Vyas et al., 2002) and dendritic atrophy in hippocampal neurons (Watanabe et al., 1992; Mitra and Sapolsky, 2008). These structural changes alter long-term potentiation and depression (Kerr et al., 1992; De Kloet et al., 1998; Yang et al., 2004; Ahmed et al., 2006) and are associated with increased anxiety and age-related cognitive impairment (Watanabe et al., 1992; Lupien et al., 1998; Revest et al., 2005). Neurons in the dorsal root ganglia (DRGs) also express GRs (DeLeón et al., 1994) but the relative level of GR expression in DRG compared to brain neurons is unknown. It is also not known whether stress/GCs can affect the structure or function of DRG neurons.

Peripheral nerve injury (e.g., axotomy or nerve ligation) activates transcriptional programs that enhance DRG neuron regenerative growth and sprouting (Smith and Skene, 1997; Neumann and Woolf, 1999). Peripheral nerve injury also causes neuropathic pain (Decosterd and Woolf, 2000; Shields et al., 2003), a condition that may develop because aberrant growth programs are elicited in sensory neurons causing sprouting into the superficial layers of the spinal cord dorsal horn (Woolf et al., 1992; Ondarza et al., 2003; Hughes et al., 2008; Zhang et al., 2015) or the nucleus gracilis and cuneatus (Persson et al., 1995; Ma and Bisby, 1998, 2000). In animal models, stress and GCs enhance nerve injury-induced pain (Wang et al., 2004; Alexander et al., 2009), although the mechanisms underlying these changes in sensation are unknown.

Our new data indicate that GCs act as circulating modifiers of sensory neuron structure and function and that DRG neurons express surprisingly high levels of GRs, even compared to hippocampal neurons, which have been considered the most GR-rich and stress-responsive neurons in the nervous system (Sapolsky et al., 1984). When DRG neurons are isolated from stressed mice and grown *in vitro*, they grow neurites that are both longer and more complex than those that grow from control DRG neurons. These growth-promoting effects of stress can be blocked using GR antagonists, and recapitulated *in vivo* by injecting synthetic GCs into naïve mice before DRG neuron harvest. Stress increases GR nuclear localization in DRG neurons but without markedly increasing the expression of regeneration-associated genes (RAGs). In fact, unlike the ability of nerve injury (e.g., conditioning lesion) to elicit RAG expression (Stam et al., 2007), stress prevents injury-induced increases in RAG expression (*Atf3*, *Ankrd1*, *Sprr1a*) while other potentially novel GR-dependent RAGs (e.g., *Gilz*, *Cebpa*) are increased by stress. Finally, we show that the axon growth-promoting effects of stress/GCs can also be identified *in vivo*. These data highlight the importance of stress and GCs as novel behavioral and circulating modifiers of sensory neuron plasticity, and bring into question whether the inflammation associated with pain should be treated with steroids.

## Materials and Methods

### Animals

Adult female C57BL/6 mice (Taconic) or *Thy1-GFP-M* mice (Feng et al., 2000) were group housed in standard cages with *ad libitum* access to food and water. Mice were maintained in a vivarium with controlled temperature (∼20°C) on a 12/12 h light/dark cycle and assigned randomly to experimental groups after a one week habituation period. All procedures were conducted by protocols approved by The Ohio State University and the University of Miami Institutional Laboratory Animal Care and Use Committee and with the guidelines of the Committee for Research and Ethical Issues of International Association for the Study of Pain.

### Western blotting

**Figure 1.**
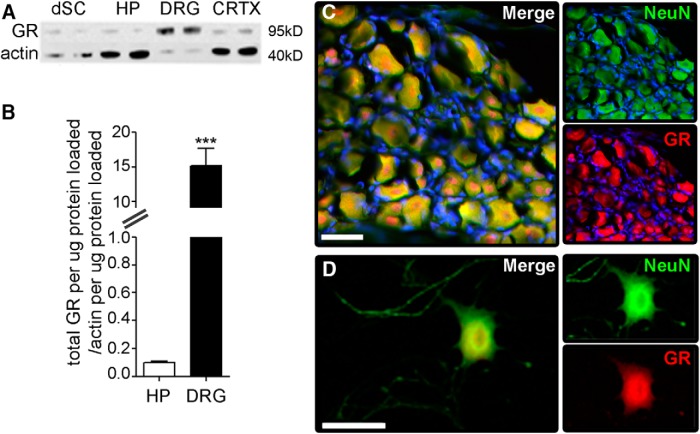
DRG neurons express high levels of GR. ***A***, GR expression was analyzed via Western blotting using protein isolated from homogenates of dorsal spinal cord (dSC), hippocampus (HP), DRG, or cerebral cortex (CRTX). ***B***, GR expression in DRG was >15-fold higher than in hippocampus (*p* < 0.0001); *N* = 4 per group, mean and SEM are shown. ***C***, Immunohistochemical staining for GR (red) in sections of whole DRG or (***D***) purified adult mouse DRG neurons *in vitro* reveal that GR is localized to neurons. NeuN is in green and DAPI in blue. Scale bar, 40 μm.

Mice were sacrificied in accordance to Institutional Animal Care and Use Committee guildelines and DRG cell lysates were prepared by homogenization in 250-μl T-PER Tissue Protein Extraction Reagent (Thermo Fisher, PI-78510) supplemented with protease and phosphatase inhibitors (Halt Cocktails, #87786; Thermo Scientific) immediately after harvest. Following centrifugation (10,000 × *g* for 5 min), protein concentration was determined using a BCA protein assay kit (Thermo Scientific, PI-23221). Samples (10 μg) were separated on 10% Bis-Tris gels and transferred to a nitrocellulose membrane in a wet-transfer apparatus (Invitrogen). After protein transfer, membranes were incubated with 5% BSA for 1 h at room temperature (RT), then with one of the following primary antibodies (1:200-1:2000): GR #PA1-511A from Thermo Scientific, RRID: AB_2236340; Actin #A1978 from Sigma-Aldrich, RRID: AB_476692, in 5% BSA at 4°C for at least 12 h, and finally with HRP-conjugated anti-rabbit IgG antibody (1:5000-1:15,000; Jackson ImmunoResearch, #111-035-046) in 5% BSA for 1 h at RT. Between incubations, the membrane was washed three times with PBS + 5% Tween for 10 min each. HRP activity was visualized using a chemiluminescent substrate (Thermo Scientific) and signal density quantified with a Kodak Image Station 4000MM Pro (Carestream Health). A ratio of signal density of phosphorylated protein to total protein was calculated. For Figure 1, the amount of DRG GR protein in 10 µg of tissue lysate exceeded the limits of a standard curve dilution produced by recombinant GR; consequently, the loading amount had to be reduced and results are evaluated on the basis of micrograms loaded. For the hippocampus, 2 µg of protein was loaded for the β-actin blot, and 20 µg for the GR blot. For the DRG, 5 µg of protein was loaded for the β-actin and 1 µg for the GR blot; *N* = 4 per condition for each experiment.

### Restraint stress protocol

We previously showed that placing mice into well-ventilated polypropylene tubes (2.8 × 9.7 cm) for 1 h elicits a transient, but significant increase in circulating corticosterone (cort; Alexander et al., 2009). This method of acute restraint stress was used throughout the experiments described in this manuscript. Nonstressed mice remained undisturbed in their home cages.

### Drugs

For *in vivo* experiments, mifepristone (RU486; 50 mg/kg, Sigma, caltalog M8046), cort (1.5 mg/kg, Sigma, catalog C174), and dexamethasone (dex; 2 mg/kg, Sigma, catalog D1756) were prepared in a sterile peanut oil vehicle (veh) and then injected in a 0.1-ml volume 1 h before cell harvest. Cells were plated and kept in Neurobasal A media for 72 h. The dose of injected cort was previously determined to reproduce stress-induced plasma cort concentrations (Alexander et al., 2009). All drugs were prepared fresh daily and delivered via intraperitoneal injection.

### *In vitr*o DRG neuron experiments

Mouse DRG were harvested after 1 h of restraint (stress) or without (nonstressed; ns), as described previously (Gensel et al., 2009). Briefly, we dissected cervical, thoracic and lumbar DRG neurons from terminally anesthetized adult mice and incubated them in dispase 2 (5 U/ml; Roche) and collagenase type 2 (200 U/ml; Worthington) for 45-60 min at 37°C in HBSS (Mediatech), followed by DNase 1 type 2 (250 µg/ml; Sigma) treatment for 5 min. Next, DRG were triturated in 0.5 ml of HBSS media through fire-polished Pasteur pipettes and spun at 3000 rpm for 3 min. The neuron-enriched pellet was resuspended in 0.1 ml of Neurobasal A media supplemented with 2% B27, 1% Glutamax, and 1% penicillin-streptomycin. Neurons were plated onto coverslips at 400 cells/coverslip, or for high-density growth analysis at 10,000 cells/coverslip. Cells assessed using automated microscopy were plated into 12-well tissue culture plates. All coverslips and tissue culture plates were pre-coated with 0.1 mg/ml of poly-L-lysine (Sigma) and 10 µg/ml of laminin (Invitrogen). DRG neurons grew for 15, 24, or 72 h at 37°C in a 5% CO_2_ humidified incubator. Different culture durations were used to interrogate different mechanisms of GC-GR-dependent growth (see Results). All DRG neurons were fixed with 2% paraformaldehyde (PFA) in PBS for subsequent immunohistochemistry. For each experiment, DRG neurons were prepared from three animals, and at least three coverslips or wells were used to quantify neurites using either Scholl Analysis or high-density neurite growth assays (below).

**Figure 2.**
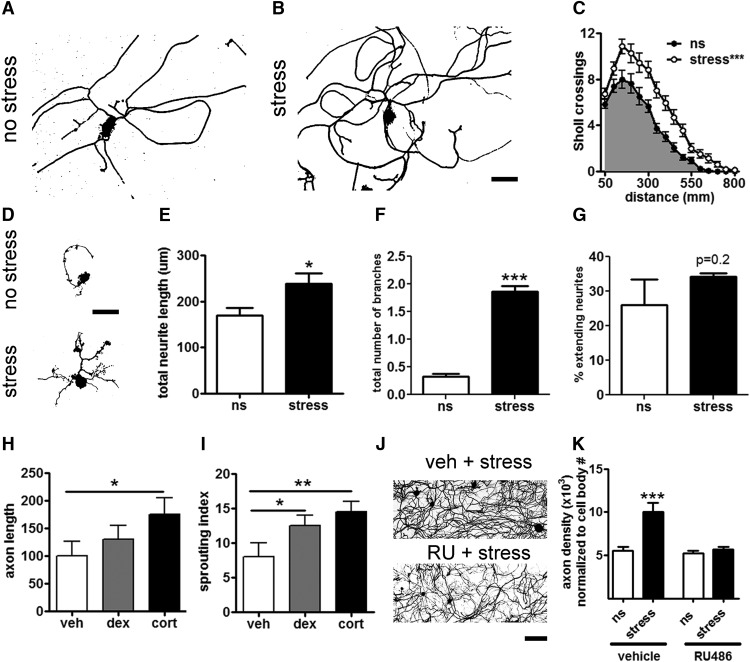
Acute stress promotes neurite growth via a GC-GR-dependent mechanism. ***A***, ***B***, DRG neurons were isolated from ns or stressed mice and grown *in vitro* for 72 h. Scale bar, 100 µm. ***C***, Stress increased neurite length. ***p < 0.0001 versus ns, one-way ANOVA. ***D-F***, Stress increases neurite growth during the “arborizing” phase of DRG neurite outgrowth. ***D***, DRG neurons from Thy1-GFP-M transgenic mice were isolated from ns adult mice or adult mice subjected to 1 h of restraint stress and grown for 15 h *in vitro*. Scale bar, 100 µm. ***E***, ***F***, Stress increased neurite total length and the number of branches per neurite, *p < 0.05, ***p < 0.0001, mean and SEM are shown. ***G***, The percentage of neurons extending neurites was unaffected by stress. All parameters were defined by the Neuronal Profiling Algorithm v4.1 (Cellomics ArrayScanXTI, Thermo Fisher). *N* ≥ 550 neurons per condition. ***H***, ***I***, Cort administration (1.5 mg/kg) 1 h before DRG harvest increased neurite elongation and sprouting measures 24 h after plating *in vitro*. ***I***, The synthetic GC, dex (2 mg/kg) increased neurite sprouting, *p < 0.05, **p < 0.01. ***J***, Representative images of high-density DRG neuron cultures (72 h *in vitro*) from vehicle-treated and RU486-treated (RU) stressed mice. Scale bar, 200 µm. ***K***, The GR antagonist, RU486 blocked stress enhanced neurite growth, ***p < 0.0001 versus ns, mean ± SEM are shown.

Rabbit polyclonal anti-β-tubulin III antibody (1:2000; Sigma, #T2200, RRID: AB_262133) or a combination of chicken anti-neurofilament (NF) 200 and 68-kDa antibodies [1:1000; Aves Labs, #NFL, NF heavy (NFH), RRID: AB_2313553 and AB_2313552] were used to visualize DRG neurons and processes. Alexa Fluor 546-conjugated goat anti-rabbit or anti-chicken IgG secondary antibodies were paired with primary antibodies to detect DRG neurons (1:1000; Invitrogen). For high-density assays at least 45% of the coverslip was randomly sampled and digitized to quantify neurites (∼100 fields; MCID 6.0 Elite). Using automatic densitometric threshold detection, neurons that were positively stained for β-tubulin III or NF were quantified then neurite density was normalized to total soma number in each field. Individual neuron neurite length in low-density cultures was quantified randomly on isolated DRG neurons after digitizing them on a light microscope followed by unbiased automated Sholl analysis (Gensel et al., 2011). Templates of concentric circles of 50-µm intervals were overlaid onto the center of a digitized DRG soma. For each neuron, densitometric thresholds were set to remove background labeling and clearly identify detailed cellular processes. The total number of objects above threshold intersecting each circle was tallied using an automated macro. The maximal ring with an intersecting process (max distance) and sum of the number of intersections (branching complexity or sprouting index) for all rings were generated for each cell and compared between groups. For automated analysis, at least 500 neurons in three technical replicates per experimental condition were examined, and each experiment was performed at least three times. In Figure 2*D-G*, individual DRG neurite length and branching were quantified using the Neuronal Profiling Algorithm (v4.1) on an ArrayScanXTI High Content Analysis Microscope (Thermo Fisher). At least 500 neurons per condition were analyzed.

### Immunohistochemistry

Mice were anesthetized with 80 mg/kg of ketamine and 10 mg/kg of xylazine before transcardial perfusion with 30 ml 0.1 M PBS (pH 7.4) followed by 100 ml of 4% PFA in PBS. DRGs were embedded in optimal cutting temperature compound (OCT; Tissue-Tek, VWR International) and frozen at -80°C; 10- to 20-μm sections were cut using a cryostat and thaw-mounted on SuperFrost Plus slides (Fisher Scientific), then stored at -20°C until use. After drying at RT, slides were rinsed with 0.1 M PBS and overlaid with blocking solution for 1 h. Sections were subsequently washed and incubated overnight with the primary antibody and appropriate secondary antibodies. We used the following primary and secondary antibodies: GR: 1:200, #PA1-511A, Thermo Fisher, RRID: AB_2236340 or sc-8992, Santa Cruz Biotechnology, RRID: AB_2155784; SCG10, 1:500, Novus Biotechnologies anti-stathmin-2, NBP1-49467, RRID: AB_10011568; ATF3, 1:500, sc-188, Santa Cruz, RRID: AB_2258513; NFH, 1:1000; Aves Labs, RRID: AB_2313552; 555 or 488-conjugated goat anti-rabbit IgG antibody, 1:500 #A11035, Invitrogen. Hoechst or DAPI was applied in the final rinse to visualize nuclei.

#### Generation of samples for qPCR analysis

A 30-s sciatic nerve crush was performed as described below. In stress + injury groups, 1 h of restraint stress (see above) was performed before injury. L3-L5 DRGs ipsilateral to the injury were removed, trimmed of their axons and then placed into Trizol for RNA extraction. A total of 500 ng of RNA was used to prepare cDNA with the RT-for-PCR cDNA kit (Clontech), and qPCR was performed as described below.

#### qRT-PCR

Gene-specific primers were used for qRT-PCR to compare expression between ns and stress groups. All primers are listed 5’-3’: *Ankrd1*: CAATGGGGCCGCAGGGGAAT, GCTTCACGCTGTTGGCCGGA; *Atf3*: AAGGGGTGATGCAACGCGCT, CGCGGGTTAGCCGATTGGCT; *Jun*: GAGTGGGAAGGACGTGGCGC, TCCATCGTTCTGGTCGCGCG; *Gap43*: GCCCCCTCCGAGGAAAAGGC, TGGCTGGGCCATCTTCAGCC; *Il6*: GCCTTCTTGGGACTGATGCT,AGTCTCCTCTCCGGACTTGTG; *Sprr1a*: CCATTGCCTTGTGCTACCAA,TCAGGAGCCCTTGAAGATGAG; *Tp53*: AGCAGGGCTCACTCCAGCTACC,GGCTGGTGATGGGGACGGGAT; *Stat3*: CGGCAGCCTGTCTGCAGAGTT,ACCAGGCAATCACAATTGGCACG; *Tsc22d3*: bhCTCGAGGGGTCGACGGAGCC,AGGTGAGCGGCACTCGGTCT; *Sgk*: GCCGGAGCGCACTGTTGTCTT,CCGATGCCCGGAAAGAGCCC; *Cebpa*: GGTACGGCGGGAACGCAACA, GAAGATGCCCCGCAGCGTGT; *Hspb2*: GGGCTCCAGTCCGGCACTTC,GCGGCGCTCGGTCATGTTCT; *Cebpb*: CGCGTTCATGCACCGCCTGC, CCAGGCAGTCGGGCTCGTAGTAG; and *Hif1a*: TCTCGGCGAAGCAAAGAGTCTG, CCCACACTGAGGTTGGTTACTGTTG. Primer sequence specificity was confirmed by performing BLAST analysis for similar sequences against known sequence databases. PCRs were conducted in triplicate using 0.001 g of cDNA/reaction and Power SYBR Green master mix (Applied Biosystems) in 0.010-ml reactions. PCR product was measured using SYBR Green fluorescence on an Applied Biosystems 7900HT system. Standard curve and melting point analyses were performed to confirm amplification efficiency and single amplified products, respectively. The ΔΔCt analysis was used to normalize gene data to 18s ribosomal RNA reference gene expression. In all cases, expression was normalized to cDNA derived from L3-L5 DRGs from ns and noninjured mice; *N* = 4 mice per group.

#### Luciferase assays

The promoters of *Cebpa*, *Cebpb*, *Cebpd*, *Stat3*, *Hif1a*, *Il6*, and *Tp53* were amplified with promoter-specific primers which also contained restriction sites so that the promoter fragments (-1000 to +300 bp from the transcription start site) could be cloned into the pLightSwitch_Prom plasmid (SwitchGear Genomics) using the In-Fusion HD Cloning Kit (Clontech). The primers are show 5’-3’: *Sgk1*: CTAGCCCGGGCTCGAGTGAACGCTTACTGGTTTTGG, TTACTTAGTTAAGCTTTGCGGCAGCGACTGCAGTAA; *Hspb2*: CTAGCCCGGGCTCGAGTGTTGATAAATTTGCATGTG, TACTTAGTTAAGCTTTTTGGACACGGAAGTCAATG; *Cebpa*: CTAGCCCGGGCTCGAGTCCCCGATTCAAGTTCACTC, TACTTAGTTAAGCTTCGTGCTCGCAGATGCCGCCC; *Cebpb*: CTAGCCCGGGCTCGAGGTCAATGGGTCGGGGGTCAG, TACTTAGTTAAGCTTGGGCTGAAGTCGATGGCGCG; *Cebpd*: CTAGCCCGGGCTCGAGGGAGCTAGGCTGCTCTGTG, TTACTTAGTTAAGCTTTGTTGAAGAGGTCGGCGAAG; *Stat3*: CTAGCCCGGGCTCGAGGTTTTTCTGCACAAGGTGTG, TACTTAGTTAAGCTTCCTGCACCCCCTTCACCTGT; *Hif1a*: CTAGCCCGGGCTCGAGCACCAGCGGACCACAGGGCT, TACTTAGTTAAGCTTCGACTTTCTCAGGCAGAAAA; *Il6*: CTAGCCCGGGCTCGAGGAGGTCCTTCTTCGATATCT, TTACTTAGTTAAGCTTGAAGTCTCCTCTCCGGACTT; and *Tp53*: CTAGCCCGGGCTCGAGACACCTCAAGCGCTGGAGAA, TACTTAGTTAAGCTTGCCAAGCTTCCATTCCGCCC. All plasmids were validated by restriction digest and sequencing. The promoter containing plasmids were individually cotransfected with wtGR, constitutively active GR (caGR) (a truncated form of GR, missing the last 250 amino acids; Addgene; pG1-GR526; plasmid #1124) or EGFP into HEK293 cells grown in a 96-well format. All transfections were conducted according to the manufacturer’s recommendations (Lipofectamine 2000, Invitrogen). HEK293 cells were cultured for 2 d *in vitro*, fluorescence was checked visually to confirm a successful transfection, and then the luciferase assay was performed following the manufacturer’s recommendations (SwitchGear Genomics). For each experiment 8 replicate wells were transfected for each condition (e.g., promoter alone, promoter + wtGR, promoter + caGR) and the values attained from the luminometer were normalized to the average of the promoter only wells to generate a fold change value; *N* = 8 replicates per plate, *N* = 3 independent experiments.

##### Quantification of GR nuclear localization

GR was visualized in DRG sections using an Axioplan 2 imaging microscope (Zeiss). All images were captured using identical exposure times, which were optimized for detecting GR in DRG from ns mice. Ganglia from L3-L5 were collected and imaged from three separate mice for each condition, and at least two sections were imaged from each ganglion. GR intensity was quantified in at least 50 randomly selected neurons using the Measure tool in ImageJ (Schneider et al., 2012). Neuron size was also measured and used to compare differences between small (10-26 µm), medium (27-42 µm) and large (43-58 µm) DRG neurons. Hoechst (Sigma) staining was used to visualize nuclei. The estimated ratio of cytoplasmic to nuclear GR intensity was calculated then these values were normalized to the average value in ns ganglia.

##### In vivo analysis of sciatic nerve regeneration

Eight-week-old female C57BL/6 mice (Taconic) were anesthetized (80 mg/kg ketamine; 5 mg/kg xylazine) then, following shaving and aseptic preparation of the right hind leg, a Dumont #5 forceps was used to crush (30-s duration) the sciatic nerve. Stressed mice underwent 1 h of restraint just before injury. The site of nerve injury was marked by charcoal. This crush duration did not produce gross motor impairment (e.g., foot drop) postoperatively. The muscle/fascia layer was pulled together and the skin was sealed with a single wound clip (Stoelting). Three days after crush injury, mice were perfused, and sciatic nerves were harvested then prepared for histology as described above. Slides were incubated overnight at 4°C with the anti-SCG10 antibody. The next day, sections were washed then incubated for 1-2 h with Alexa Fluor 555- or 488-conjugated goat anti-rabbit IgG antibodies. Nerves were imaged with identical exposure times (Axioplan 2 imaging microscope, Zeiss). A photo montage was created in Photoshop then SCG10 intensity was quantified using ImageJ as described previously (Abe et al., 2010; Shin et al., 2012; Cho et al., 2013). Briefly, the width of each nerve was measured every 500 µm then averaged. The raw intensity every 100 µm was measured then normalized by dividing by average nerve width. The crush site was identified as the point with the highest intensity as in (Shin et al., 2014) . The regeneration index was calculated as the farthest point from the site of injury where pixel intensity was at least 50% of the intensity at the crush site (Abe et al., 2010); *N* = 4 nerves/mouse; *N* = 3 mice/group.

A separate cohort of mice was used for 3-dimensional (3D) imaging and axon quantification. The same 30-s unilateral sciatic nerve crush injury was performed on eight-week-old female Thy1-GFP-M (The Jackson Laboratory, stock #007788) mice (Feng et al., 2000). These mice were perfused at 14 d postinjury (dpi). After perfusion, sciatic nerves were rapidly removed and postfixed overnight at 4°C, followed by a rinse in 100 mM PBS. Sciatic nerves were cleared using 50% tetrahydrofuran (THF) for 25 min, 80% THF for 30 min, 100% THF 2Xs for 30 min, 100% dichloromethane for 20 min, and finally in Dibenzyl ether (Sigma-Aldrich) for 15 min (Ertürk et al., 2012). The whole sciatic nerve was imaged using a fluorescence light sheet microscope (LaVision BioTec Ultramicroscope). Axon quantification was performed with an unbiased approach, using the Spots algorithm in Bitplane Imaris Scientific 3D/4D Image Processing Software (Oxford Instruments) at 0.5-mm intervals from the crush site to the distal end of the nerve. The number of “spots” at each interval was entered for each mouse (*N* = 3/group) then compared between groups (stress vs no-stress).

##### Statistics

*In vitro* and *in vivo* neurite/axon growth was analyzed using one- or two-way repeated measures analysis of variance with Bonferroni or Fisher *post hoc* analyses. Student’s *t* test was used to analyze data from histologic experiments comprising only two groups. A ratio *t* test for paired data were applied to Western blot analyses to compare fold change between groups. An α-level of *p* < 0.05 was used to indicate statistical significance.

## Results

### Sensory neurons express high levels of GRs

Stress activates the hypothalamic-pituitary-adrenal axis leading to an increase in the synthesis and release of GCs into the circulation. High levels of circulating GCs directly regulate gene transcription by binding to GRs. Hippocampal neurons express high levels of GRs, and GC-GR interactions profoundly affect hippocampal neuron structure/function (Sapolsky et al., 1984; Joëls et al., 2003; Fitzsimons et al., 2013). In sensory neurons, however, neither the relative abundance of GRs nor the effects of GC-GR interactions are known. We used Western blotting to compare the relative amounts of GRs in spinal cord, hippocampus, DRG and cortex. Although GRs were found in all regions, GR levels in DRG were ∼15-fold greater than in the hippocampus (Fig. 1*A*,*B*). Whole DRGs or purified adult DRG neurons labeled with anti-GR antibodies confirmed GR localization predominantly in neurons (Fig. 1*C*,*D*). These data indicate that DRG neurons express surprisingly high levels of GRs, suggesting that circulating or exogenous GCs may exert potent effects on DRG neurons through GRs.

### Stress induces sensory neurite growth

Stress and GCs induce structural plasticity in hippocampal neurons with corresponding behavioral changes that manifest as impaired cognition and anxiety. Previously, we found that stress and GCs enhance nerve injury-induced pain (Alexander et al., 2009). To test whether the enhanced nociceptive effects of stress and GCs were also associated with structural changes in sensory neurons, adult DRG neurons were isolated from stressed or ns mice, then morphologic features of neurite growth were analyzed. Immediately after stress, DRG neurons were isolated and placed in culture for 72 h, a time in which most DRG neurons have extended neurites. Neurite length and complexity were analyzed using unbiased automated Sholl analysis (Gensel et al., 2011). Stress consistently and significantly increased the complexity (number of Sholl crossings; indicative of arborization) and overall length of neurites *in vitro* (Fig. 2*A-C*).

### Stress promotes neurite growth via GC-GR-dependent regulation of genes that are constitutively expressed in sensory neurons

Previous data indicate that neurite outgrowth from uninjured adult DRG occurs in two phases (Smith and Skene, 1997). In the early phase (∼12-16 h after plating), small numbers of neurons exhibit highly branched arborizing neurites but limited extension of any individual neurite. Genes that are constitutively expressed *in vivo* by DRG neurons regulate this early (arborizing) phase of neurite growth. In the second phase (≥24 h), most DRG neurons develop the capacity for rapid and sustained axonal elongation. This later and distinct phase of axon growth requires transcription of new genes including various canonical RAGs (e.g., GAP43; Smith and Skene, 1997; Frey et al., 2000; Cafferty et al., 2001; Bonilla et al., 2002; Campbell et al., 2005; Qiu et al., 2005; Seijffers et al., 2007).

Because data in Figure 2*A-C* were generated from DRG neurons cultured for 72 h, it is not possible to know whether stress affected the constitutively expressed genes that control early neurite growth or the later RAG-dependent growth programs. Thus, in a follow-up experiment, DRG neurons were isolated from ns or stressed mice, then grown in culture for 15 h, a time preceding the delayed induction of RAG expression (Smith and Skene, 1997). After 15 h, neurons from ns mice extended short neurites (<200 µm) with few arborizations (Fig. 2*D*). In contrast, stress increased the overall length and branching complexity of DRG neurites (Fig. 2*E*,*F*); the total number of DRG neurons with multibranched neurites tripled, and the total number of branches/neuron increased 6-fold. Stress did not affect the percentage of neurons that extended neurites (Fig. 2*G*), indicating that stress acts on existing neurite outgrowth pathways to induce neurons to extend neurites at a faster rate.

To determine if these acute effects of stress could be mediated by GCs, in lieu of stress, naïve mice were injected intraperitoneally with cort (1.5 mg/kg) or a synthetic GC (dex; 2 mg/kg) 1 h before DRG harvest. Neurons were evaluated at 24 h, well before the marked increases in RAG expression that occurs in cultured DRG neurons (Smith and Skene, 1997). Structural plasticity increases similar to those caused by stress were seen with either cort or dex; cort increased neurite length and complexity, whereas dex increased only neurite sprouting (Fig. 2*H*,*I*).

To determine if the stress/GC effects were mediated via binding to GR *in vivo*, mice were injected with the GR-antagonist RU486 (50 mg/kg) before stress. High-density DRG neuron cultures were used since they require less effort to prepare and analyze, and because stress and cort were found to increase the density of neurites in high-density cultures (data not shown). As shown in Figure 2*J*,*K*, acute GR block with RU486 prevented the increase in neurite outgrowth caused by stress.

Combined, these data indicate that stress enhances axon outgrowth from sensory neurons in part via GC-GR-dependent interactions. These changes occur early (<24 h), before axotomy induced transcriptional programs, and thus might work through GC-GR-dependent transcription pathways.

### Stress promotes DRG neurite growth independently of RAGs

**Figure 3.**
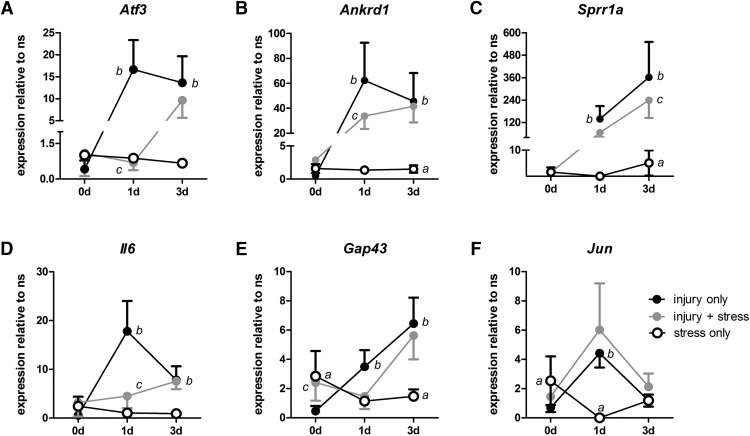
Stress represses injury-induced RAG expression. ***A-F***, RAG expression in DRGs was evaluated by qRT-PCR from ns mice and compared to expression in DRGs isolated immediately (0) or 1 or 3 d after: stress only (open circles), injury only (black circles), or when restraint stress was applied 1 h before injury (gray circles). Stress alone increases *Gap43* (***E***) and *Jun* (***F***) expression at 0 d, represses *Jun* at 1 d (***F***), and increases *Ankrd1* (***B***), *Sprr1a* (***C***), and *Gap43* (***E***) at 3 d. Stress compared to ns, ^a^*p* < 0.05-0.0005. ***A-F***, Injury significantly increases expression of all RAGs at 1 d and *Atf3* (***A***), *Ankrd1* (***B***), *Sprr1a* (***C***), *IL6* (***D***), and *Gap43* (***E***) at 3 d. Injury compared to ns mice, ^b^*p* < 0.0001. ***A***, ***B***, ***D***, Stress-reduced injury induction of *Atf3* (***A***), *Ankrd1* (***G***), and *Il6* (***I***) at 1 d; *Sprr1a* (***H***) at 3 d; and increased *Gap43* at 0 d. Injury compared to injury + stress, ^c^*p* < 0.001-0.0001, one-way ANOVA with Bonferroni or Fisher’s *post hoc* tests. Mean ± SD are shown, *N* = 4 per group.

The ability of stress to induce vigorous neurite growth in DRG neurons is reminiscent of the “conditioning effects” of a peripheral nerve injury. Indeed, DRG neurons that have been previously injured have an increased ability to initiate regenerative axon growth and this is dependent on new gene transcription (i.e., RAGs; Smith and Skene, 1997; Frey et al., 2000; Cafferty et al., 2001; Bonilla et al., 2002; Campbell et al., 2005; Qiu et al., 2005; Seijffers et al., 2007). To test whether expression of canonical RAGs that are normally induced by nerve injury (e.g., *Atf3*, *Ankrd1*, *Sprr1a*, *Il-6*, *Gap43*, and *Jun*) also are upregulated by stress, mRNA was prepared from DRG neurons that were isolated from mice immediately following stress (0 d), or 1 or 3 d after exposing them to 1 h of restraint stress (Fig. 3*A-F*, open circles). Stress increased the expression of *Ankrd1*, *Sprr1a*, *Gap43*, and *Jun* mRNA; however, the magnitude and temporal progression of these changes varied by gene. Both *Gap43* and *Jun* mRNA were immediately increased (relative to ns mice, 0 d; Fig. 3*E*,*F*, open circles). Conversely, *Ankrd1* and *Sprr1a* increased but not until 3 d after stress. Although *Jun* expression increased immediately after stress, *Jun* expression was significantly reduced by 1 d (relative to ns), a finding consistent with GR-dependent regulation of *Jun* (Wei et al., 1998). *Jun* expression returned to baseline by 3 d (Fig. 3*F*). Overall, stress had only a mild effect on inducing expression of a subset of RAGs. By comparison, RAG expression was markedly increased 1 and 3 d after a peripheral nerve injury in ns mice (Fig. 3*A-F*, black circles).

These data indicate that stress increases neuronal expression of a subset of RAGs, but that these effects are delayed and are orders of magnitude lower than the effects of a peripheral nerve injury. Thus, GR-dependent induction of canonical RAGs alone is unlikely to explain the consistent and substantial increase in neurite growth caused by stress (Fig. 2). In fact, based on timing and the relative magnitude of changes in RAG expression, stress/GCs and nerve injury appear to regulate neurite growth via independent transcriptional mechanisms.

To test this hypothesis, acute stress was applied for 1 h before a sciatic nerve crush. One or three days later, DRGs were dissected and gene expression was analyzed using qPCR. Using this approach, gene expression changes caused by stress and injury will occur together and interact in DRG neurons *in vivo*. Remarkably, restraint stress (1 h) before nerve injury caused a partial or complete block of the injury-induced increase in RAG expression (Fig. 3*A-F*, gray circles). Only nerve injury-induced changes in Jun expression were unaffected by stress (Fig. 3*F*). When compared to the effects of injury alone, stress significantly reduced the expression of most injury-induced RAGs; *Atf3*, *Ankrd1*, and *Il6* levels were significantly reduced in DRG neurons from stressed mice as compared to injury only mice (Fig. 3*A*,*B*,*D*, compare gray circles with black circles, *C*).

**Figure 4.**
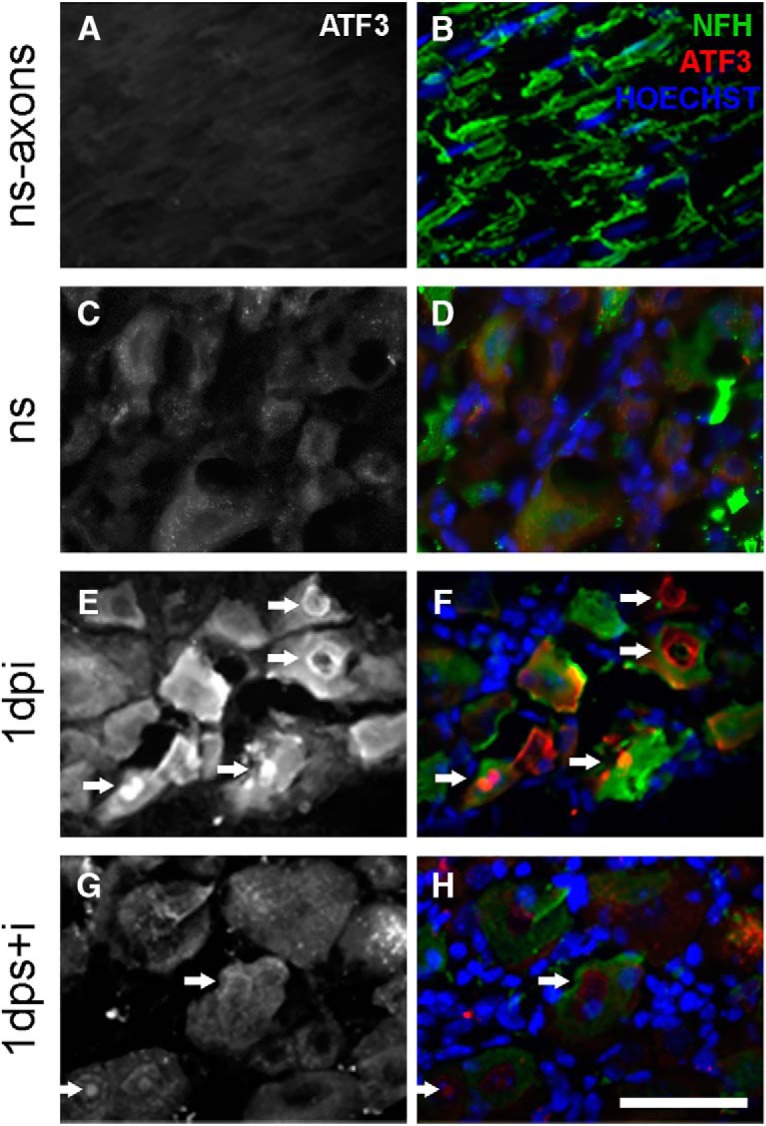
Stress reduces ATF3 immunostaining in DRG neurons. ***A***, ***B***, ATF3 was nearly undetectable in sensory neuron axons and (***C***, ***D***), DRG neurons from ns mice. ***E***, ***F***, DRG collected 1 dpi showed increased ATF3 immunoreactivity compared to DRGs collected from mice receiving stress + injury, ***G***, ***H***, Arrowheads indicate ATF3 nuclear immunoreactivity. ***B***, ***D***, ***F***, ***H***, NFH is shown in green, ATF3 in red, and Hoechst in blue. Scale bar, 100 µm.

The effects of stress on reducing nerve injury-induced gene expression were most prominent for *Atf3*, with near complete inhibition of *Atf3* upregulation at 1 dpi (Fig. 3*A*). To determine if reduced *Atf3* expression was matched with a reduction in ATF3 protein, anti-ATF3 antibodies were used to label sections of DRG (Fig. 4). No ATF3 was detected in axons or neuronal somata from uninjured ns mice (Fig. 4*A-D*). As expected, nerve injury increased somal ATF3 at 1 dpi (Fig. 4*E*,*F*). In contrast, minimal ATF3 was evident in DRG tissue sections from injured mice that had been stressed before nerve injury (Fig. 4*G*,*H*, arrowheads).

Together, data in Figures 2-4 indicate that stress increases neurite growth in a GC-GR-dependent manner; however, these effects occur without inducing the expression of canonical RAGs. In fact, stress blocks or suppresses the expression of RAGs that are normally increased by nerve injury.

### Stress increases nuclear translocation of the GR

**Figure 5.**
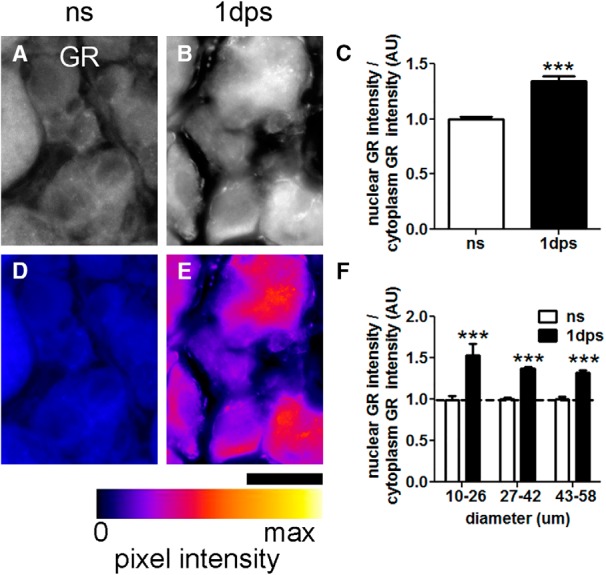
Stress increases GR nuclear localization in DRG neurons. ***A***, ***B***, GR immunofluorescence in DRG neurons from ns and stressed (1 d ps) mice. ***D***, ***E***, Images in ***A***, ***B*** converted to an intensity map overlay using the Fire lookup table (ImageJ). Fire LUT pixel intensity map key show colors corresponding to pixels from 0 to max intensity. Scale bar, 25 µm. ***C***, Stress increases nuclear GR pixel intensity in DRG compared to ns mice. *N* ≥ 50 neurons (see Materials and Methods). One-way ANOVA followed by Bonferroni *post hoc* tests, compared to ns. Mean and SEM are shown, ***p < 0.0001. ***F***, Cell areas were divided into three groups to represent small (10-26 µm), medium (27-42 µm), and large (43-58 µm) DRG neurons. Increases in GR nuclear localization were consistent across different sized DRG neurons.

A stress-induced change in gene transcription should be associated with translocation of GR from the cytoplasm to the nucleus. To determine if this occurs in stressed DRG neurons, mice were subjected to acute stress for 1 h then 1 d later, DRGs were removed and stained with antibodies that label GR (Fig. 5*A-E*). In naive mice, GR labeling intensity was low (blue coloring; also see Materials and Methods; Fig. 5*A-D*). In contrast, stress markedly increased the ratio of nuclear to cytoplasmic GR intensity. These data indicate that stress promotes nuclear translocation of GR, and supports the notion that axon growth programs are affected by GR-dependent regulation of gene transcription.

Because phenotypically and functionally distinct classes of sensory neurons exist within DRGs (Usoskin et al., 2015; Li et al., 2015), stress or GCs may not uniformly affect nuclear translocation of GRs in these varied DRG neuron subtypes. To test this hypothesis, the mean GR signal intensities in Figure 5*C* were binned as a function of neuron diameter [e.g., small (10-26 µm), medium (27-42 µm), or large (43-58 µm)], a classification scheme that roughly correlates with division of DRG subtype by Trk family receptor expression (i.e., TrkA are small, TrkB are medium, TrkC are large diameter; McMahon et al., 1994; Usoskin et al., 2015). As shown in Figure 5*F*, GR nuclear intensity increased across all neuronal size classes, suggesting that GC/GR-dependent changes in gene expression affect all sensory neurons.

### Predicted transcriptional regulation of RAGs by the GR

To identify genes in sensory neurons that are directly activated or repressed by GRs, we first used GeneGo to analyze a list of >400 genes that had already been identified in injured DRG neurons (Seijffers et al., 2007; Stam et al., 2007; Hajji et al., 2008; Wu et al., 2008; Moore et al., 2009; Michaelevski et al., 2010). Using this approach, 28 transcription factors (TFs) were identified whose expression is directly increased or decreased by GR transcriptional activity or binding (Table 1). Next, we evaluated the list of >400 genes to identify genes regulated by these TFs. This approach identified an additional 100 genes (Table 2). In addition to known targets, several potentially novel GR targets were identified by scanning the putative promoter regions (-1000 bp, +300 bp from the transcription start site) of genes for predicted GR-response elements (GREs), i.e., short sequences bound by GRs (http://jaspar.genereg.net). GREs were identified in the promoters of several TFs and RAGs expressed in DRG neurons (Table 3), suggesting that GRs can exert a surprisingly large and diverse effect on the transcriptional machinery that controls sensory neuron growth and function.

**Table 1.** TFs associated with regeneration whose expression is also impacted by the GR (transcriptionally activated/repressed by GR)

Transcription factor	Effect of GR on transcription
C/EBPα	+
C/EBPβ	+
COUP-TFII	+
c-Rel (NF-κB subunit)	+
HIF1A	+
IRF1	+
NURR1	+
p73	+
PPAR-γ	+
SF1	+
STAT3	+
STAT5	+
STAT5A	+
STAT5B	+
TBP	+
c-Jun/c-Fos	-
c-Rel (NF-κB subunit)	-
E2A	-
NF-AT2(NFATC1)	-
NF-κB	-
NF-κB1 (p50)	-
p53	-
p73	-
PIT1	-
RelA (p65 NF-κB subunit)	-
SMAD3	-
STAT6	-
T-bet	-
ZNF307	-

Interactions were determined using MetaCore software and were identified in mouse, rat, and human.

**Table 2.** GR regulated TFs in column A (interaction from) and the targeted downstream RAGS in column B (interaction to)

Interaction from	Interaction to	Interaction effect	Interaction type	Organisms
c-Rel (NF-κB subunit)	c-Myb	+	TR	Homo;Mus;Rattus
c-Rel (NF-κB subunit)	STAT1	u	TR	Homo;Mus;Rattus
c-Rel (NF-κB subunit)	STAT5A	u	TR	Homo;Mus;Rattus
c-Rel (NF-κB subunit)	IL-6	+	TR	Homo;Mus;Rattus
C/EBPα	RNF12	u	TR	Homo;Mus;Rattus
C/EBPα	c-Fos	+	TR	Homo;Mus;Rattus
C/EBPβ	STAT6	+	B	Homo;Mus;Rattus
C/EBPβ	b-Myb	u	TR	Homo;Mus;Rattus
C/EBPβ	LTBP2	u	TR	Homo;Mus;Rattus
C/EBPβ	HNF1-α	+	B	Homo;Mus;Rattus
C/EBPβ	HNF1-β	+	B	Homo;Mus;Rattus
C/EBPβ	PAX2	u	TR	Homo;Mus;Rattus
C/EBPβ	c-Jun	+	B	Homo;Mus;Rattus
C/EBPβ	Ghrelin	u	TR	Homo;Mus;Rattus
C/EBPβ	IL-6	+	TR	Homo;Mus;Rattus
C/EBPβ	c-Fos	+	TR	Homo;Mus;Rattus
c-Jun/c-Fos	c-Fos	u	TR	Homo;Mus;Rattus
COUP-TFII	HNF1-β	+	TR	Homo;Mus;Rattus
E2A	ZNF143	u	TR	Homo;Mus;Rattus
E2A	c-Fos	u	TR	Homo;Mus;Rattus
GCR-α	STAT5A	+	B	Homo;Mus;Rattus
GCR-α	TBP	+	B	Homo;Mus;Rattus
GCR-α	STAT6	-	B	Homo;Mus;Rattus
GCR-α	c-Jun/c-Fos	-	B	Homo;Mus;Rattus
GCR-α	SMAD3	-	B	Homo;Mus;Rattus
GCR-α	IL-6	-	TR	Homo;Mus;Rattus
GCR-α	ZNF167	u	TR	Homo;Mus;Rattus
HIF1A	IL-6	+	TR	Homo;Mus;Rattus
HIF1A	SMAD3	+	B	Homo;Mus;Rattus
HIF1A	Mxi1	+	TR	Homo;Mus;Rattus
HIF1A	RNF183	u	TR	Homo;Mus;Rattus
HIF1A	c-Jun	+	B	Homo;Mus;Rattus
HIF1A	Junctin	+	TR	Homo;Mus;Rattus
IRF1	RNF168	u	TR	Homo;Mus
IRF1	c-Myb	-	TR	Homo;Mus;Rattus
IRF1	RNF12	u	TR	Homo;Mus;Rattus
IRF1	c-Jun	+	B	Homo;Mus;Rattus
NF-AT2(NFATC1)	LTBP3	+	TR	Homo;Mus;Rattus
NF-κB	PAX2	u	TR	Homo;Mus;Rattus
NF-κB	PTBP1	u	TR	Homo;Mus;Rattus
NF-κB	FosB	u	TR	Homo;Mus;Rattus
NF-κB	JunB	u	TR	Homo;Mus;Rattus
NF-κB	Ghrelin	u	TR	Homo;Mus;Rattus
NF-κB	Junctin	u	TR	Homo;Mus;Rattus
NF-κB	STAT5A	u	TR	Homo;Mus;Rattus
NF-κB	IL-6	+	TR	Homo;Mus;Rattus
NF-κB	SMAD3	+	B	Homo;Mus;Rattus
NF-κB1 (p50)	c-Fos	-	B	Homo;Mus;Rattus
NF-κB1 (p50)	c-Myb	+	TR	Homo;Mus;Rattus
NF-κB1 (p50)	JunB	u	TR	Homo;Mus;Rattus
NF-κB1 (p50)	IL-6	+	TR	Homo;Mus;Rattus
NF-κB1 (p50)	FosB	u	TR	Homo;Mus;Rattus
NF-κB1 (p50)	c-Jun	-	B	Homo;Mus;Rattus
Oct1	JunD	u	TR	Homo;Mus;Rattus
Oct1	IL-6	u	TR	Homo;Mus;Rattus
Oct1	FosB	u	TR	Homo;Mus;Rattus
Oct1	STAT4	u	TR	Homo;Mus;Rattus
Oct1	LTBP3	u	TR	Homo;Mus;Rattus
Oct1	HNF1-β	u	TR	Homo;Mus;Rattus
Oct1	HNF1-α	+	B	Homo;Mus;Rattus
p53	RNF10	u	TR	Homo;Mus;Rattus
p53	STAT4	u	TR	Homo;Mus;Rattus
p53	b-Myb	-	B	Homo;Mus;Rattus
p53	TBP	-	B	Homo;Mus;Rattus
p53	A-Myb	u	TR	Homo;Mus;Rattus
p53	c-Myb	-	B	Homo;Mus;Rattus
p53	Large T antigen (SV40)	+	B	Homo;Mus;Rattus
p53	STAT5A	-	Unspecified	Homo;Mus;Rattus
p53	c-Fos	u	TR	Homo;Mus;Rattus
p53	IL-6	-	TR	Homo;Mus;Rattus
p73	ZNF143	+	B	Homo;Mus;Rattus
p73	STAT1	+	TR	Homo;Mus;Rattus
PIT1	c-Fos	+	TR	Homo;Mus;Rattus
PPAR-γ	SMAD3	-	B	Homo;Mus;Rattus
RelA (p65 NF-κB subunit)	STAT6	u	TR	Homo;Mus;Rattus
RelA (p65 NF-κB subunit)	RhoA	+	TR	Homo;Mus;Rattus
RelA (p65 NF-κB subunit)	JunB	+	TR	Homo;Mus;Rattus
RelA (p65 NF-κB subunit)	STAT5A	+	TR	Homo;Mus;Rattus
RelA (p65 NF-κB subunit)	FosB	+	TR	Homo;Mus;Rattus
RelA (p65 NF-κB subunit)	IL-6	+	TR	Homo;Mus;Rattus
RelA (p65 NF-κB subunit)	ZNF143	u	TR	Homo;Mus;Rattus
SMAD3	c-Jun	+	TR	Homo;Mus;Rattus
SMAD3	LTBP3	+	TR	Homo;Mus;Rattus
SMAD3	JunB	+	TR	Homo;Mus;Rattus
SMAD3	c-Jun/c-Fos	+	B	Homo;Mus;Rattus
STAT3	ZNF148	-	B	Homo;Mus;Rattus
STAT3	STAT1	u	TR	Homo;Mus;Rattus
STAT3	IL-6	+	TR	Homo;Mus;Rattus
STAT3	JunB	+	TR	Homo;Mus;Rattus
STAT3	LTBP3	u	TR	Homo;Mus;Rattus
STAT3	STAT2	u	TR	Homo;Mus;Rattus
STAT3	c-Fos	+	TR	Homo;Mus;Rattus
STAT5	c-Fos	+	TR	Homo;Mus;Rattus
STAT5	STAT1	-	C	Homo;Mus;Rattus
STAT5A	c-Fos	+	TR	Homo;Mus;Rattus
STAT5B	STAT1	-	C	Homo;Mus;Rattus
STAT6	STAT1	-	C	Homo;Mus;Rattus
STAT6	RhoA	+	TR	Homo;Mus;Rattus
T-bet	STAT1	+	TR	Homo;Mus;Rattus
T-bet	SETBP1	+	TR	Homo;Mus;Rattus

The effect of the interaction on target gene expression is shown in column C, and the type of interaction is in column D. The organism this interaction was reported in is listed in column E. + indicates activation; - indicates inhibition; u indicates unspecified interaction. TR indicates transcription regulation; B indicates binding; C indicates competition. Homo: *Home sapiens*, Mus: *Mus musculus*; Rattus: *Rattus norvegicus*.

**Table 3.** GR (*Nr3c1*) TF binding sites predicted by the JASPAR database (Wasserman and Sandelin, 2004)

Gene	Chromosome	Strand	Number of sites	Relative score	Position in promoter	Predicted binding site sequence
Ankrd1	19	-	2	0.8	723	CAAAGCAACACTTCCCAG
				0.82	738	AGAAATAGGATGTCCCAA
Atf3	1	-	3	0.75	731	GAAGGCACATTTTCCTGA
				0.75	1022	AAAAACGTTTTGTGGTTG
				0.8	1163	GAGATCAAAGCGTCCTCT
Cebp-α	7	+	3	0.77	216	AAAAAGAAAGTTTTCCAG
				0.75	336	GGGACCCTGTAGTTCTAG
				0.75	412	GAGAAAAAGACGCACAAT
Cebp-β	2	+	0			
Cebp-δ	16	+	2	0.75	102	GGACAGATGATTTTCTTG
				0.76	348	AGAAACAGCAAGATGCTA
Gap43	16	-	3	0.76	29	GAAAAAAAAATTTTTTTT
				0.75	381	AGAGAGAGAATGTGCGTG
				0.75	1209	AGAAAGAATCACAACTGT
Hif1a	12	+	3	0.75	672	CAGTTCCTCATGTCGTGG
				0.77	758	AAAATCATAATGTAAATA
				0.76	879	AAAAAAAAAACTTACGTG
Hsbp1(Hsp27)	9	+	2	0.75	600	CAAAACACTCACTCCAGA
				0.87	655	AAGAAAATTTTTTCCTAA
IL-6	5	+	1	0.84	352	AGAAACAACTGGTCCTGA
Jun	4	-	5	0.75	331	GGTGACATCATGGGCTAT
				0.79	419	CAGAGAAGAATCTTCTAG
				0.79	841	CAGATCATTCAGCCCTTT
				0.77	1003	CGAAACTAAACTTCCAAG
				0.77	1101	GAGAATAAAGTGTTGTGC
NFκBia	12	-	1	0.81	349	AGGACGAGCCAGTTCTTT
p21(Cdkn1a)	17	+	6	0.77	131	GACAGCATCCTTTCCTTC
				0.81	184	GAAATGATCGCGTTCTGG
				0.83	233	GGGAAAAAAATCTCCAGA
				0.77	616	AGGCAAACACTGTACCAA
				0.81	685	GAAAAGAGTTAGTCCTTC
				0.75	740	CAGCTCTAACTGTACTGT
p53	11	+	1	0.85	403	GAAAACAGTCTTTACAGA
Sgk1	10	+	3	0.76	579	GAGACCATTCACTGCTAC
				0.77	605	AGGCACATTATTTTATTT
				0.75	766	AAGAAAAACCAATTCAAA
Sprr1a	3	-	0			
Stat3	11	-	2	0.79	987	GGAAATGGCAAGTACTGT
				0.76	1069	GGAAACAAGTTGGTCAAA

For each gene, the promoter was defined as -1000 and +300 bp from the transcription start site.

**Figure 6.**
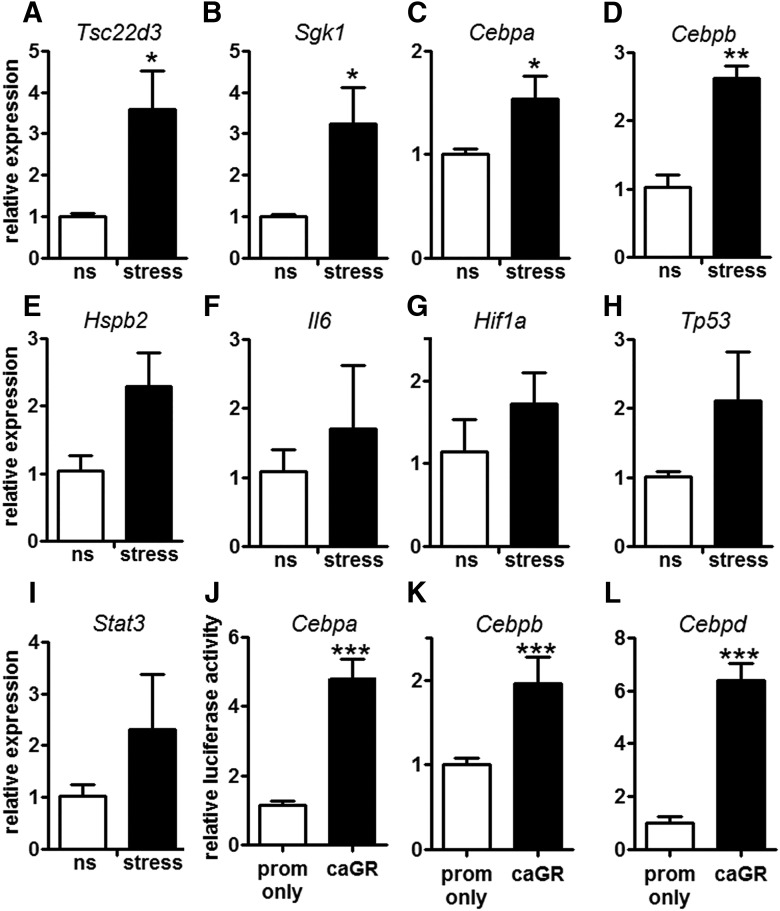
Stress and GRs activate *Cebp* TF expression. ***A-D***, Stress (1 h) increases *Tsc22d3* (***A***), *Sgk1* (***B***), *Cebpa* (***C***), and *Cebpb* (***D***) expression in DRG. *Hspb2* (***E***), *Il6* (***F***), *Hif1a* (***G***), *Tp53* (***H***), and *Stat3* (***I***) expression were unaffected; *N* = 3. ***J-L***, caGR increased luciferase expression from the *Cebpa* (***J***), *Cebpb* (***K***), and *Cebpd* (***L***) promoter; *N* = 8 replicates for each condition per experiment; *N* = 3 experiments. ***p < 0.0005, one-way ANOVA followed by Dunnett’s multiple comparison test with the promoter only as the control. Mean and SEM are shown.

To validate the *in silico* analysis above, qPCR was used to quantify a subset of genes/TFs in DRG neurons from control (ns) or stressed mice. Expression of *Tsc22d3* (also known as Gilz), a stress-induced gene with GREs in its promoter region, was analyzed as a positive control gene (Fig. 6*A*; Riccardi et al., 2000). Stress increased the expression of *Tsc22d3* and also *Sgk*, *Cebpa*, and *Cebpb* (Fig. 6*A-D*). Other RAGs, many of which have been described as potent transcriptional regulators of axon regeneration including *Hspb2* (also known as Hsp27), *Il-6*, *Hif1a*, *Tp53* (also known as p53), and *Stat3*, were also affected by stress, although gene expression changes were highly variable and were not significantly different from controls (Fig. 6*E-I*).

To show that GC-GR interactions could play a role in inducing expression of these genes, we tested the ability of caGR to drive luciferase expression from the promoters of *Cebpa*, *Cebpb*, *Cebpd*, *Stat3*, *Hif1a*, *Sgk1*, *Hspb2*, *Il-6*, and *Tp53.* Luciferase activity was not changed when *Hif1a*, *Sgk1*, *Hspb2*, or *Tp53* were coexpressed with caGR and inconsistent luciferase activity was observed for *Stat3* and *Il-6* (data not shown). In contrast, transfection with caGR consistently increased luciferase activity from the promoters of *Cebpa*, *Cebpb*, and *Cebpd* (Fig. 6*J-L*). These data indicate that GR can bind to the promoters of *Cebpa* and *Cebpd* (Table 3), presumably through the predicted GREs, and that GRs can activate transcription of these genes.

### Stress increases peripheral nerve axon regeneration

**Figure 7.**
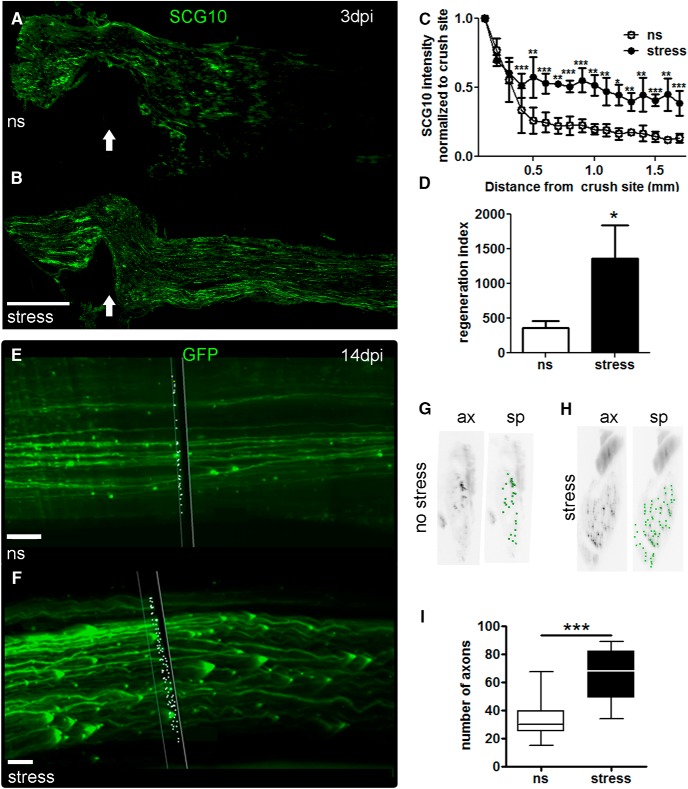
Stress enhances regeneration of injured sensory axons *in vivo*. ***A***, ***B***, Representative longitudinal sections of sciatic nerves from mice that received a crush injury alone or 1 h of restraint followed by crush injury. Nerves were immunostained with SCG10. Arrow indicates crush site. Scale bar, 500 µm. ***C***, Stress increased SCG10+ intensity distal to the crush site by 3 dpi. Results are expressed as mean ± SEM. **p < 0.001, ***p < 0.0001 for two-way ANOVA for distance and condition; *N* = 3 per group, four sections per animal. ***D***, Stress increases the regeneration index. Results are expressed as mean ± SD. *p < 0.01, unpaired *t* test. ***E-I***, Representative images of cleared intact sciatic nerve distal to the crush site in ns (***E***) and stressed (***F***) mice. The plane through the nerve (black box with white outline) and the white dots show the results of the Spots algorithm (Bitplane) used for axon quantification. Scale bar, 70 µm. ***G***, ***H***, A single cross section view through the intact sciatic nerve rendered in black and white for easy viewing (left panel; axons: ax). Green dots indicate axon cross sections and show the outcome of the spots algorithm (sp). ***I***, Summary statistics for the experiment are shown in a box plot for the average number of axons measured every 0.5 mm distal to the crush site in the sciatic nerve in ns and stressed mice. Average, 33 ns versus 64 stressed, ***p < 0.0001; minimum, 15 ns versus 34 stressed; median, 30 ns versus 63 stressed; maximum, 68 ns versus 89 stressed; *N* = 3 per group.

The experiments above indicate that stress increases sensory axon growth via GC-GR-dependent mechanisms. To determine if stress/GCs cause similar effects on axon growth *in vivo*, peripheral nerve regeneration was evaluated in ns and stressed mice that received a sciatic nerve crush. Sections of injured sciatic nerves (3 dpi) from stressed or ns mice were labeled with SCG10, a protein that is preferentially expressed in regenerating sensory axons (Shin et al., 2014). SCG10 intensity was significantly higher compared to ns mice (Fig. 7*A-C*). Also, the axon regeneration index, the point along the nerve at which the SCG10 labeling was half the intensity found at the crush site (Abe et al., 2010), was increased by stress (Fig. 7*D*).

To eliminate any variability or unintended sampling bias that might be associated with immunostaining or tissue cutting/processing, we also evaluated axon regeneration using unbiased 3D microscopy. *Thy1*-GFP-M transgenic mice in which ∼10% of neurons, including DRG neurons, express GFP (Feng et al., 2000) were divided into stress or no-stress groups; all mice then received a sciatic nerve crush. Whole sciatic nerves were collected and cleared (3DISCO; Ertürk et al., 2012) and all axons were quantified using fluorescence light sheet microscopy and Imaris 3D Visualization Software (Bitplane, Oxford Instruments; Fig. 7*E*,*F*; Movies 1, 2; Ertürk et al., 2012; Renier et al., 2014). Axon profiles in the distal nerve segment were quantified at 14 dpi, a time when injured axons and myelin debris are normally cleared from the injury site (Vargas et al., 2010). This provides a “clean” background for assessing total numbers of regenerating axons. Using this approach, we found that stress increased the overall number of GFP+ axons ∼2-fold throughout the 5-cm distance examined distal to the crush site when compared with injured nerves from ns mice (Fig. 7*G-I*). Together, these data show that acute stress augments regenerative growth of injured peripheral nerves *in vivo*.

10.1523/ENEURO.0246-17.2017.video.1 Chromeless Video Player V4Issue4-0246-Movie1 Movie 1. Thy1-GFPM sciatic nerve in a ns mouse 14 d after crush injury. Green fibers are axons distal to the crush site. The box through the image shows an area quantified using the spots algorithm (Imaris), and white dots show the results of the algorithm, indicating counted axons.

10.1523/ENEURO.0246-17.2017.video.2 Chromeless Video Player V4Issue4-0246-Movie2 Movie 2. Thy1-GFPM sciatic nerve in a stressed mouse 14 d after injury crush injury. Green fibers are axons distal to the crush site. The box through the image shows an area quantified using the spots algorithm (Imaris) and white dots show the results of the algorithm, indicating counted axons.

## Discussion

Novel data in this report show that stress and GCs enhance the sprouting and regenerative growth of adult sensory axons, *in vitro* and *in vivo*. Using *in silico* modeling strategies, we identified several TFs and RAGs with GC-response elements and show that some of these genes are regulated by stress via GR-dependent mechanisms.

In the brain, stress and GCs enhance structural plasticity (e.g., dendrite growth/retraction), neurogenesis, memory, and neuronal excitability (Imperato et al., 1991; Kaufer et al., 1998; Kirby et al., 2013). Because hippocampal neurons express high basal levels of GRs, they are thought to be uniquely responsive to circulating GCs (Fuxe et al., 1985; Reul and De Kloet, 1985). However, our data indicate that sensory neurons also express high GR levels, more so than hippocampal neurons, and are exquisitely sensitive to stress and circulating GCs.

The axon growth promoting effects of stress and GCs are reminiscent of the effects of injuring the peripheral branch of DRG sensory neurons (McQuarrie and Grafstein, 1973; Lasek et al., 1984; Smith and Skene, 1997). These “conditioning” lesions promote axon growth in part by activating developmental growth programs and transcription of RAGs (McQuarrie and Grafstein, 1973; Bonilla et al., 2002; Qiu et al., 2005). Because GRs are TFs that are activated by stress (or circulating GCs), we hypothesized that they would similarly enhance RAG expression, providing an explanation for the ability of stress/GCs to augment sensory axon growth *in vitro* and *in vivo*. Instead, we found that although stress enhances axon outgrowth through GC-GR-dependent mechanisms, these effects likely occur rapidly (immediately poststress) and do not involve transcriptional regulation of traditional RAGs. Indeed, stress only modestly increased expression of a subset of RAGs and such changes typically took 1-3 d. Other traditional RAGs (e.g., *Jun*, *Il6*, *Atf3*) were repressed. GR repression of *Il6* and *Atf3* is consistent with previous studies of *Il6* in COS cells and *Atf3* in macrophages (Verhoog et al., 2011; Chinenov et al., 2014). Moreover, when paired with a conditioning lesion, stress partially inhibited or delayed the injury-evoked increases in RAG expression. Thus, GRs likely power sensory axon growth by controlling the transcription of genes that are constitutively expressed *in vivo* by DRG neurons and not traditional RAGs. Using *in silico* analyses, we identified several candidate genes (Tables 1-3) and demonstrate that the CCAAT/enhancer binding proteins (*Cebp*) are at least one gene family that is positively regulated by GC-GR binding.

*Cebps* are transcriptional regulators of cellular differentiation, terminal function and responses to inflammation and tissue injury (Poli, 1998; Ménard et al., 2002; Calella et al., 2007). In the nervous system, *Cebpb* expression increases in regenerating facial motor neurons and may control the expression of other genes necessary for axon growth (Nadeau et al., 2005). Loss of *Cebpd* function in mice delays regeneration of injured peripheral nerve (Lopez de Heredia and Magoulas, 2013). Although we did not prove that *Cebps* are responsible for the axon growth promoting effects of stress, our data do show that *Cebpa* and *Cebpb* expression are induced by acute stress and that GC-GR interactions drive expression of *Cebp-a*, *-b*, and *-d*. Although these data implicate *Cebps* as regulators of stress-induced neurite growth, additional studies are needed to unequivocally prove a causal role for these TFs in regulating stress-induced axon plasticity.

The current experiments focused on the effects of GC-GR interactions in adult sensory neurons. Other neurons and glia also express GRs, and in response to injury or stress, GCs undoubtedly affect structure and function in these cells. As an example, after spinal cord injury basal GR expression increases in spinal neurons, but it is not known how GR activation influences neural plasticity (Ferrini et al., 1993; Wang et al., 2004). GR activation in microglia and astrocytes can induce or repress cell cycle, proliferation, and inflammatory processes (Crossin et al., 1997; Hwang et al., 2006; Frank et al., 2010; Carter et al., 2012); and GC-GR interactions influence Schwann cell proliferation (Neuberger et al., 1994), which is required for efficient peripheral nerve regeneration (Hall, 1986). In future studies, conditional targeting of GRs will reveal the range of mechanisms and cell types that underlie stress-enhanced growth and plasticity after nerve injury.

The current data may have broad clinical implications, since stress is ubiquitous and synthetic corticosteroids are widely used as anti-inflammatory agents to treat various diseases in animals and humans. Perhaps most notable are the consequences that the present data could have for the diagnosis and treatment of neuropathic pain. Consider that in animal models of nerve injury or inflammatory pain (Wang et al., 2004; Khasar et al., 2005, 2008; Alexander et al., 2009), stress and GCs exacerbate pain behaviors and these effects are reversed by GR inhibitors. Stress can also elicit or exacerbate pain in human disease (Ashkinazi and Vershinina, 1999; Turner et al., 2002; Greco et al., 2004; Nicholson and Martelli, 2004; DeLeo, 2006). The mechanisms underlying chronic pain are complex and incompletely understood; however, a number of publications indicate that nerve injury causes functional reorganization of injured sensory neurons, with sprouting of sensory axons, mostly nociceptive afferents, into the superficial layers of the spinal cord dorsal horn (Woolf et al., 1992; Okamoto et al., 2001; Ondarza et al., 2003; Hughes et al., 2008; Woodbury et al., 2008; Zhang et al., 2015). Perhaps stress and GCs exacerbate this nerve-injury induced phenomenon of axonal plasticity within the superficial dorsal horn. We also cannot rule out the possibility that stress-induced increases in GCs could enhance pain respones by increasing axonal sprouting (“plasticity”) in other areas of the CNS involved in sensory processing (e.g., nucleus cuneatus and gracilis; Persson et al., 1995; Ma and Bisby, 1998, 2000). Overall, our data highlight the importance of stress and GCs as novel behavioral and circulating modifiers of sensory neuron plasticity and should prompt new discussion about whether the inflammation associated with pain should be treated with steroids.

## Synthesis

Reviewing Editor: Mary Heinricher, Oregon Health & Science University Decisions are customarily a result of the Reviewing Editor and the peer reviewers coming together and discussing their recommendations until a consensus is reached. When revisions are invited, a fact-based synthesis statement explaining their decision and outlining what is needed to prepare a revision will be listed below. Note: If this manuscript was transferred from JNeurosci and a decision was made to accept the manuscript without peer review, a brief statement to this effect will instead be what is listed below.

The authors present intriguing data implicating stress and circulating glucocorticoids in plasticity in the dorsal root ganglion after a nerve injury. Although much work needs to be done to fully understand the mechanisms underlying this effect, the overall effect is well-documented here, and should stimulate further study in this area.
